# Traffic-Related Air Pollution, Noise at School, and Behavioral Problems in Barcelona Schoolchildren: A Cross-Sectional Study

**DOI:** 10.1289/ehp.1409449

**Published:** 2015-08-04

**Authors:** Joan Forns, Payam Dadvand, Maria Foraster, Mar Alvarez-Pedrerol, Ioar Rivas, Mònica López-Vicente, Elisabet Suades-Gonzalez, Raquel Garcia-Esteban, Mikel Esnaola, Marta Cirach, James Grellier, Xavier Basagaña, Xavier Querol, Mònica Guxens, Mark J. Nieuwenhuijsen, Jordi Sunyer

**Affiliations:** 1 Centre for Research in Environmental Epidemiology (CREAL), Barcelona, Catalonia, Spain; 2 Universitat Pompeu Fabra, Barcelona, Catalonia, Spain; 3 CIBER Epidemiologia y Salud Pública (CIBERESP), Barcelona, Catalonia, Spain; 4 Department of Genes and Environment, Division of Epidemiology, Norwegian Institute of Public Health, Oslo, Norway; 5 Swiss Tropical and Public Health Institute, Basel, Switzerland; 6 University of Basel, Basel, Switzerland; 7 Institute of Environmental Assessment and Water Research (IDAEA-CSIC), Barcelona, Catalonia, Spain; 8 IMIM (Hospital del Mar Medical Research Institute), Barcelona, Catalonia, Spain; 9 Department of Epidemiology and Biostatistics, Imperial College London, United Kingdom; 10 Department of Child and Adolescent Psychiatry/Psychology, Erasmus University Medical Centre–Sophia Children’s Hospital, Rotterdam, the Netherlands

## Abstract

**Background:**

The available evidence of the effects of air pollution and noise on behavioral development is limited, and it overlooks exposure at schools, where children spend a considerable amount of time.

**Objective:**

We aimed to investigate the associations of exposure to traffic-related air pollutants (TRAPs) and noise at school on behavioral development of schoolchildren.

**Methods:**

We evaluated children 7–11 years of age in Barcelona (Catalonia, Spain) during 2012–2013 within the BREATHE project. Indoor and outdoor concentrations of elemental carbon (EC), black carbon (BC), and nitrogen dioxide (NO2) were measured at schools in two separate 1-week campaigns. In one campaign we also measured noise levels inside classrooms. Parents filled out the strengths and difficulties questionnaire (SDQ) to assess child behavioral development, while teachers completed the attention deficit/hyperactivity disorder criteria of the DSM-IV (ADHD-DSM-IV) list to assess specific ADHD symptomatology. Negative binomial mixed-effects models were used to estimate associations between the exposures and behavioral development scores.

**Results:**

Interquartile range (IQR) increases in indoor and outdoor EC, BC, and NO2 concentrations were positively associated with SDQ total difficulties scores (suggesting more frequent behavioral problems) in adjusted multivariate models, whereas noise was significantly associated with ADHD-DSM-IV scores.

**Conclusion:**

In our study population of 7- to 11-year-old children residing in Barcelona, exposure to TRAPs at school was associated with increased behavioral problems in schoolchildren. Noise exposure at school was associated with more ADHD symptoms.

**Citation:**

Forns J, Dadvand P, Foraster M, Alvarez-Pedrerol M, Rivas I, López-Vicente M, Suades-Gonzalez E, Garcia-Esteban R, Esnaola M, Cirach M, Grellier J, Basagaña X, Querol X, Guxens M, Nieuwenhuijsen MJ, Sunyer J. 2016. Traffic-related air pollution, noise at school, and behavioral problems in Barcelona schoolchildren: a cross-sectional study. Environ Health Perspect 124:529–535; http://dx.doi.org/10.1289/ehp.1409449

## Introduction

There is a growing body of evidence on associations between pre- and postnatal exposure to traffic-related air pollutants (TRAPs) and adverse impacts on cognitive development in children (Block and Calderón-Garcidueñas 2009; Guxens and Sunyer 2012; Guxens et al. 2012; Suglia et al. 2008). Air pollution is considered a suspected cause of developmental neurotoxicity (Grandjean and Landrigan 2014). In this context, TRAPs may also affect behavioral development. The available evidence for such an effect is limited and mostly focused on attention deficit/hyperactivity disorder (ADHD) symptomatology, suggesting a positive association between prenatal (Perera et al. 2006, 2012) and early-life exposure to TRAPs (Newman et al. 2013) and ADHD. Only one study has reported a positive cross-sectional association between ADHD and TRAPs exposure during childhood (Siddique et al. 2011).

Like air pollution, noise is a ubiquitous environmental pollutant, generated mostly by traffic in urban areas (Moudon 2009). Noise has been associated with adverse impacts on quality of life and health, including mental health (Basner et al. 2014). It has been suggested that children are vulnerable to noise because of their reduced ability to manage environmental stressors (Ben-Shlomo and Kuh 2002). Therefore, efforts have been made to understand the impact of noise exposure on cognitive development (Stansfeld et al. 2005). Some studies have suggested that the negative impact of road traffic and aircraft noise at school and aircraft on cognitive development in children may exceed that of air pollution (Clark et al. 2012; Hygge et al. 2002; van Kempen et al. 2010, 2012). The available body of evidence on the associations of noise exposure with child behavioral development is limited, but associations have been reported between increased hyperactive symptomatology and both traffic noise at home (Tiesler et al. 2013) and aircraft noise at school (Crombie et al. 2011; Stansfeld et al. 2005).

Most of the literature in this field is focused on children’s residential exposures to these pollutants. However, children spend long periods of time at school, where levels of TRAPs and noise peak during the day (Kim et al. 2002; Ning et al. 2007). Teaching requires a quiet environment in classrooms. According to the World Health Organization’s recommendation, a noise level < 35 dB in classrooms is considered optimal. However, it is not unlikely that the noise exceeds this level in some schools (Berglund et al. 1999). The physical activity of children at school may be increased due to physical education classes, sports, or play activities, and their breathing rates and dose of inhaled pollutants to the lungs may increase during school hours (McConnell et al. 2010). In the present study we aimed to investigate the association between levels of TRAPs and noise at school and behavioral developmental in schoolchildren.

## Methods

*Study setting.* This study was carried out as part of the BRain dEvelopment and Air polluTion ultrafine particles in scHool childrEn (BREATHE) project, which aims to study associations between air pollution and neuropsychological development of schoolchildren. Schools were selected based on modeled levels of traffic-related nitrogen dioxide (NO_2_) to achieve maximum contrast in TRAP levels (Wang et al. 2013). Thirty-six of the 416 schools in Barcelona were selected. Three additional schools in an adjacent municipality, Sant Cugat del Vallès, were included in BREATHE (39 schools in total). All participating schools were similar to the remaining schools in Barcelona in terms of the socioeconomic vulnerability index (0.66 vs. 0.62, *p* = 0.20) and NO_2_ levels (51.5 vs. 50.9 μg/m^3^, *p* = 0.81).

All families of children without special needs from these 39 schools in the 2nd, 3rd, and 4th primary grades were invited to participate in the study by letter and/or presentations in schools. A total of 2,897 (59%) enrolled in the study. All children had been at the school for > 6 months (and 98% for > 1 year) before the beginning of the study. We observed similar participation rates across classes. Participation rates were similar across classes (62%, 61%, and 62% for 2nd, 3rd, and 4th grades, respectively, *p* = 0.96). Participation rates were similar according to the school vulnerability index (62% and 61% for higher and lower school vulnerability index, respectively, *p* = 0.326), but not between public (52%) and private schools (66%) (*p* < 0.01). Parents or legal guardians of all participating children gave their written informed consent as approved by the IMIM-Parc Salut Mar Ethical Committee (No. 2010/41221/I).

*Exposure.* School air pollution measurement. For each school, TRAP levels were measured twice during 1-week periods separated by 6 months, in the warm and cold periods of the year 2012. In each campaign week, we measured TRAP levels in two schools simultaneously: one located in an area with high levels of pollution, and one in an area with low levels. Levels of TRAPs were measured simultaneously indoors in a single classroom and outdoors in the schoolyard. We selected measured elemental carbon (EC), black carbon (BC), and NO_2_, given their high correlation to road traffic emissions in Barcelona (Amato et al. 2014; Reche et al. 2014; Rivas et al. 2014). Initially, we also considered concentrations of traffic-related particulate matter with aerodynamic diameter ≤ 2.5 μm (PM_2.5_) [composed of organic particles from motor exhaust, EC, and metals from brake wear (copper, antimony, tin, and iron)] as another measure of exposure to TRAPs. However, given the high correlation between EC and traffic-related PM_2.5_ concentration (Spearman rho = 0.93) and the consistency of our results for these two measures, only the results for EC are included in this manuscript. EC levels were determined based on chemical analysis of daily 8-hr PM_2.5_ samples collected by high volume (30 m^3^/hr) MCV samplers. Real-time concentrations of BC were measured using the MicroAeth AE51 aerosol monitor (AethLabs). Weekly averaged NO_2_ concentrations were measured using Gradko Environmental passive dosimeters.

Annual outdoor and indoor school levels were obtained by averaging the results of the two campaigns. Because different schools were monitored in different weeks during each campaign period, we adjusted the levels of each TRAP for the weekly average level of that TRAP (during the corresponding sampling week for each school) measured by a background monitoring station in Barcelona to remove temporal fluctuation in background TRAP levels from our analyses (Rivas et al. 2014).

Noise measurement. Traffic noise in the classroom (hereafter, noise) was selected as a surrogate of noise exposure because children spend most of their time at school in the classroom, and also because susceptibility to the cognitive effects of traffic noise might be greater during teaching sessions (Basner et al. 2014). Data were obtained from comprehensive noise measurements taken during the second 1-week air pollution sampling period. Noise measurements were conducted during the second week of the air pollution sampling period in a single classroom for each school. We installed the noise monitoring instrument in a classroom next to the classroom where the TRAPs measurement was performed, to avoid the noise from the TRAPs monitors while having comparable measures in terms of orientation and floor. Three consecutive 10-min measurements of equivalent sound pressure levels [dB(A)] at different distributed locations within the classroom were performed over two consecutive days, following recommended protocols from the Catalan Government: Decret 176/2009 de la Generalitat, Annex 7.II.B, ISO 1996, and ISO 140-4 (Catalan Government 2009) using a calibrated SC-160 sound level meter [CESVA Inc.; ± 1.0 dB tolerance (type 2), range, 30–137 dB]. Because we aimed to register traffic and background noise levels, any unusual sounds were deleted and measurements were done before children arrived (before 0900 hours). For robustness, we averaged the 30-min measurements from the 2 consecutive days, though they showed high reproducibility.

*Behavioral development.* General behavioral development was assessed using the Strengths and Difficulties Questionnaire (SDQ) (Goodman 1997), which was filled out by parents. The SDQ comprises five separate subscales, each including 5 questions (25 questions in total) covering different behavioral aspects including emotional symptoms, conduct problems, hyperactivity/inattention, peer relationship problems, and prosocial behavior. Each question can be rated on a 3-point Likert scale [not true (0), somewhat true (1), and certainly true (2)], and each subscale can therefore be scored between 0 and 10. A total difficulties score (ranging from 0 to 40) is then generated by summing the scores for all subscales except the prosocial behavior scale, with higher scores indicating more behavioral problems. The prosocial subscale is considered a behavioral strength. We inverted the original distribution of the prosocial behavior scale to harmonize the interpretation of the subscales (higher score meaning less prosocial behavior).

Specific ADHD symptomatology was reported by teachers filling out the ADHD Criteria of *Diagnostic and Statistical Manual of Mental Disorders, Fourth Edition* (ADHD-DSM-IV) list (American Psychiatric Association 2002). ADHD-DSM-IV consists of 18 symptoms categorized into two separate groups: inattention (9 symptoms) and hyperactivity/impulsivity (9 symptoms). Each ADHD symptom is rated on a 4-point scale (0 = never or rarely, 1 = sometimes, 2 = often, or 3 = very often), and subscales can therefore be scored from 0 to 27 and the global ADHD symptomatology score can range from 0 to 54, where higher numbers indicate more problems.

*Other variables.* Sociodemographic data including child age and sex (male and female) and maternal education (primary, secondary, and university) were collected by the BREATHE baseline questionnaire, which was filled out by parents. In the same questionnaire, parents were asked whether their children had been clinically diagnosed with ADHD by a medical doctor. We also included a question regarding traffic noise annoyance at home using an 11-point scale question developed by the International Commission on the Biological Effects of Noise (ICBEN) (Fields et al. 2001). For each home and school address, we extracted the Urban Vulnerability Index, which is a measure of neighborhood socioeconomic status (SES) at the census tract level (median area of 0.08 km^2^ for the study area). This index is based on 21 indicators of urban vulnerability grouped into four themes that have been developed based on the 2001 Spanish census data: sociodemographic vulnerability (5 indicators), socioeconomic vulnerability (6 indicators), housing vulnerability (5 indicators), and subjective perception of vulnerability (5 indicators). Annual air pollution concentrations (BC) at the participants’ home addresses were estimated by temporally adjusted land-use regression (LUR) models developed as part of the European Study of Cohorts for Air Pollution Effects (ESCAPE) project (Beelen et al. 2013; Eeftens et al. 2012). To explore the comparability of levels of TRAPs measured by BREATHE monitors at schools and levels of TRAPs estimated using ESCAPE LUR models, we applied ESCAPE LUR models to estimated NO_2_ and BC outdoor levels at schools during the corresponding BREATHE sampling weeks for that school. We observed Spearman’s correlation coefficients of 0.81 and 0.75 for NO_2_ and BC, respectively, measured by BREATHE monitors and estimated by ESCAPE LUR models.

*Statistical analysis.* Main analyses. We estimated associations of indoor and outdoor levels of TRAPs (EC and NO_2_) and noise on the total difficulties score from SDQ and on the total ADHD symptomatology from the ADHD-DSM-IV list (one at a time) in two sets of models: single-exposure models, using noise and TRAP levels as the exposure one at a time in separate models; and multi-exposure models, which included each TRAP and noise together in the same model. Negative binomial mixed-effects models (including school and teacher as random effects for SDQ and ADHD-DSM-IV list, respectively) were used to address the multilevel nature of the data and to account for overdispersion of the outcomes. Negative binomial model models the ratio of the mean score among exposed and nonexposed. The results are presented as adjusted mean ratios (aMR) with corresponding 95% confidence intervals (CIs). In all of these models, the exposure estimate was reported for one interquartile range (IQR) increase in exposure levels—for example, a ratio of mean score of 1.15 for an IQR increase in EC exposure meant that those with an EC exposure at the 75th percentile had a 15% higher mean score, compared with those with an EC exposure at the 25th percentile. The analyses were adjusted for a number of covariates identified *a priori*: child’s sex (male/female), child’s age (continuous, years), BC concentrations at home (continuous, micrograms per cubic meter), traffic noise annoyance at home (continuous, ranging from 0 to 10, from the less to the highest annoyance), home tobacco use (no, yes but not inside home, yes), and indicators of SES at the individual level [maternal education (primary or less, secondary, or high educational level), and the Urban Vulnerability Index at home address (continuous variable ranging from 0 to 1, with 1 indicating the highest level of deprivation)] and the area level [Urban Vulnerability Index for the child’s school and the type of school (public/private)].

Further and sensitivity analyses. Because of the rather high correlation between TRAPs and noise, we evaluated the potential multicollinearity using the multilevel variance inflation factors (MVIFs). In addition, we tested for potential effect modification by noise [low noise (< 35 dB); high noise (≥ 35 dB)] in the association between TRAPs and the total difficulties score from SDQ and on the total ADHD symptomatology from the ADHD-DSM-IV list. We included the product term between TRAPs and the effect modifier, considering *p* < 0.05 as the cut-off for interaction.

We further adjusted (one at a time) our main analyses for maternal occupation (self-employed, employee, housewife, student, on work leave, unemployed, or unknown), paternal education (primary or less, secondary, or higher educational level), siblings at birth (no/yes), smoking during pregnancy (no/yes), alcohol consumption during pregnancy (no/yes), and duration of breastfeeding (no breastfeeding, < 1 week, 1 week–3 months, 3–6 months, and > 12 months) to evaluate the robustness of our findings to inclusion of more SES-related or biologically relevant covariates. We repeated the main models, stratifying by high- and low-polluted schools based on the previously modeled NO_2_ to explore a potential variation in the associations across these schools. ESCAPE LUR models (Beelen et al. 2013) were applied to estimate the annual (2009) NO_2_ levels at schools and schools were categorized as high- or low-polluted if their NO_2_ levels were below or above the median of all the schools participating in the BREATHE project.

We also estimated associations between TRAPs and noise at school with the five SDQ subscales and the two ADHD-DSM-IV list subscales. We further estimated associations with BC at each child’s home address using estimates from ESCAPE LUR models (Eeftens et al. 2012).

Finally, we repeated the main analysis but restricted it to those children attending the same school from the age of ≤ 3 years [*n* = 2,192 (81%)], to explore possible chronic associations of the reported associations.

Statistical analyses were carried out with Stata 12 (StataCorp, College Station, TX, USA) and R (R Core Team 2013).

## Results

Sociodemographic characteristics of the study sample are presented in Table 1. Six percent of children were reported as clinically diagnosed with ADHD. The median scores for SDQ total difficulties was 8 and for SDQ subscales ranged from 1 to 3 (Table 2). For ADHD symptomatology, the median score was 5 for total, 3 for inattention, and 1 for hyperactivity/impulsivity (Table 2).

**Table 1:** Characteristics of the study population of the BREATHE project.

Variable	*n *(%)
Child’s age (years) (mean ± SD)	8.55 ± 0.88
Child’s grade
2nd	1,076 (37.4)
3rd	1,028 (35.7)
4th	772 (26.8)
Sex
Male	1,446 (50.3)
Female	1,430 (49.7)
Clinical diagnosis of ADHD by medical doctor	
No	2,544 (93.5)
Yes	176 (6.5)
Maternal education
Primary or less	346 (12.7)
Secondary	781 (28.7)
University	1,596 (58.6)
Urban Vulnerability Index at home address
Less deprived	1,061 (37.2)
Normal deprived	1,301 (45.6)
High deprived	491 (17.2)
Type of school
Public	1,031 (35.9)
Private	1,845 (64.2)

**Table 2:** Univariate description of the outcomes (at individual level) and the exposures (at school level).

Variable	Minimum	25th percentile	Median	75th percentile	Maximum
Outcomes at individual level
SDQ (*n* = 2,714)
Total difficulties score	0	4	8	11	32
Emotion symptoms	0	1	2	3	10
Conduct problems	0	0	2	3	10
Hyperactivity/inattention	0	2	3	5	10
Peer relationship problems	0	0	1	2	9
Prosocial behavior	0	0	1	2	9
ADHD-DSM-IV (*n* = 2,805)
ADHD symptomatology	0	1	5	12	54
Inattentive symptomatology	0	0	3	8	27
Hyperactive symptomatology	0	0	1	4	27
Exposure at school level (*n* = 39)
Indoor (μg/m^3^)
EC	0.44	0.77	1.19	1.77	3.47
BC	0.60	1.00	1.37	1.86	3.30
NO_2_	11.47	21.58	29.82	42.60	65.65
Outdoor (μg/m^3^)
EC	0.58	1.02	1.33	1.88	3.90
BC	0.65	1.13	1.33	1.75	3.38
NO_2_	25.92	35.10	48.46	57.37	84.55
Noise
Indoor (dB)	28.80	34.00	38.00	42.00	51.10
Exposure at home address-individual level (*n* = 2,805)
BC (μg/m^3^)	0.86	2.17	2.63	3.06	5.45

Regarding the exposure, EC and BC concentrations were almost the same indoors and outdoors, whereas the outdoor concentrations of NO_2_ were higher than the indoor concentrations (Table 2). The median (IQR) indoor noise was 38 (8) dB. The Spearman correlation coefficient between school TRAPs levels ranged between 0.71 and 0.97 (Table 3). Noise showed moderate correlations with school TRAPs levels with correlation coefficients ranging between 0.38 and 0.45 (Table 3).

**Table 3:** Spearman correlation coefficients between TRAPs at school (μg/m^3^) and the noise at school (dB).

	Indoor			Outdoor			Noise
	EC	BC	NO_2_	EC	BC	NO_2_	
Indoor
EC	—
BC	0.97*	—
NO_2_	0.78*	0.74*	—
Outdoor
EC	0.88*	0.88*	0.71*	—
BC	0.89*	0.87*	0.78*	0.97*	—
NO_2_	0.84*	0.82*	0.76*	0.85*	0.84*	—
Noise	0.45*	0.40*	0.38*	0.39*	0.42*	0.42*	—
*p < 0.05							

Median SDQ total difficulties scores were higher for children attending public versus private schools (9.03 and 7.80, respectively, *p* = 0.017) (see Supplemental Material, Table S1). We found higher scores in SDQ for those children attending schools with medium vulnerability index values (median SDQ score = 9.17) than for children attending schools with low (median SDQ score = 7.77) and high (median SDQ score = 8.58) values. ADHD symptomatology scores were not significantly associated with the type of school or school vulnerability index.

*Main analysis.* In single-exposure models (Table 4), higher levels of TRAPs were generally associated with higher SDQ total difficulties scores with statistically significant associations for an IQR increase in indoor EC (IQR = 1.01 μg/m^3^) (aMR = 1.07; 95% CI: 1.01, 1.12), outdoor EC (IQR = 0.85 μg/m^3^) (aMR = 1.07; 95% CI: 1.03, 1.12), and outdoor NO_2_ (IQR = 22.26 μg/m^3^) (aMR = 1.07; 95% CI: 1.01, 1.14). Noise was not associated with the SDQ total difficulties score (Table 4). In the multi-exposure models, we observed the same pattern of results. Because of the high correlation between EC and BC (Spearman rho = 0.97), and because associations for BC were identical to those obtained for EC, we only present associations for EC.

**Table 4:** Adjusted mean ratios (95% CIs) of SDQ total difficulties score and ADHD symptomatology (from ADHD-DSM-IV) for TRAPs exposure at school (μg/m^3^) and noise at school (dB) as continuous variable (based on an IQR increase).

Variable	EC and noise		NO_2_ and noise	
	EC indoor IQR = 1.01 μg/m^3 ^EC outdoor IQR = 0.86 μg/m^3^	Noise IQR = 7.60 dB	NO_2_ indoor IQR = 21.01 μg/m^3 ^NO_2_ outdoor IQR = 22.26 μg/m^3^	Noise IQR = 7.60 dB
Total difficulties score (SDQ)^*a*^
Indoor
Single-exposure	1.07 (1.01, 1.12)*	1.01 (0.96, 1.07)	1.02 (0.96, 1.08)	1.01 (0.96, 1.07)
Multi-exposure	1.08 (1.02, 1.14)**	0.98 (0.92, 1.04)	1.02 (0.95, 1.10)	1.01 (0.94, 1.07)
Outdoor
Single-exposure	1.07 (1.03, 1.12)**	1.01 (0.96, 1.07)	1.07 (1.01, 1.14)*	1.01 (0.96, 1.07)
Multi-exposure	1.08 (1.03, 1.13)**	0.97 (0.92, 1.03)	1.08 (1.01, 1.16)*	0.98 (0.92, 1.04)
ADHD symptomatology (DSM-IV)^*b*^
Indoor
Single-exposure	0.96 (0.89, 1.03)	1.22 (1.11, 1.34)**	1.08 (0.99, 1.17)	1.22 (1.11, 1.34)**
Multi-exposure	0.89 (0.82, 0.96)*	1.29 (1.18, 1.43)**	0.98 (0.89, 1.08)	1.24 (1.12, 1.38)**
Outdoor
Single-exposure	0.99 (0.93, 1.07)	1.22 (1.11, 1.34)**	1.03 (0.94, 1.13)	1.22 (1.11, 1.34)**
Multi-exposure	0.94 (0.87, 1.01)	1.27 (1.15, 1.40)**	0.94 (0.85, 1.04)	1.26 (1.14, 1.39)**
Single-exposure models including TRAPs (EC and NO_2_) were adjusted for child’s sex, child’s age, maternal education, urban vulnerability index at home address, air pollution (BC) at home, home tobacco use, urban vulnerability index at school, and type of school. Single-exposure models including noise were adjusted for child’s sex, child’s age, maternal education, urban vulnerability index at home address, traffic noise annoyance at home, home tobacco use, urban vulnerability index at school, and type of school. Multi-exposure models including TRAPs and noise were adjusted for child’s sex, child’s age, maternal education, urban vulnerability index at home address, air pollution (BC) at home, traffic noise annoyance at home, home tobacco use, urban vulnerability index at school, and type of school. ^***a***^Including school as random effect. ^***b***^Including teacher as random effect. *p < 0.05. **p < 0.001.				

We observed higher ADHD symptomatology in association with an IQR increase in noise (IQR = 7.60 dB)(aMR = 1.22; 95% CI: 1.11, 1.34) in the single-exposure models (Table 4). None of the TRAPs were statistically associated with ADHD symptomatology in the single-exposure models. In the multi-exposure models, we observed a stronger positive association between noise (an IQR increase) and ADHD symptomatology (aMRs = 1.24–1.29). The associations for TRAPs remained nonsignificant in the multi-exposure models except for the association with indoor EC, for which a statistically significant inverse relationship was found (Table 4). Results for BC were the same as the results obtained for EC (data not shown).

*Further and sensitivity analyses.* The MVIFs for TRAPs and noise when combined in two-exposure models for both outcomes ranged between 1.29 and 1.45 (as a rule of thumb, MVIF > 10 is suggestive for multicollinearity) (see Supplemental Material, Table S2). The interaction between noise and TRAP was statistically significant only for the association between indoor NO_2_ and ADHD symptomatology [aMR = 0.93; 95% CI: 0.72, 1.19 vs. 1.11; 95% CI: 1.02, 1.22 when stratified by low (< 35 dB) and high noise, respectively, *p* = 0.03]; otherwise, associations with TRAPs were similar when stratified by noise (see Supplemental Material, Table S3).

Our findings did not change meaningfully after further adjusting for SES-related or biologically relevant covariates (data not shown). When we stratified the main analyses by high- and low-polluted schools based on the previous measure of NO_2_, we observed similar results (data not shown).

We found similar positive associations for most SDQ subscales, particularly for hyperactivity/inattention, peer relationship problems, and prosocial behavior in association with EC indoor and outdoor and NO_2_ outdoor (see Supplemental Material, Figure S1). Similarly, the results for the ADHD-DSM-IV subscales were similar to those for the overall ADHD symptomatology scores, with positive associations between noise and both inattentiveness and hyperactivity (see Supplemental Material, Figure S2). Noise was associated with increased symptomatology of inattentiveness and hyperactivity. Concentrations of BC at each child’s home address using estimates from ESCAPE LUR models were not associated with either behavioral problems or with ADHD symptomatology (see Supplemental Material, Table S4). When we restricted the main analysis to those children attending the same school since ≤ 3 years of age *n* = 2,192 (81%), we observed similar results to those reported for the main analysis (see Supplemental Material, Table S5).

## Discussion

To our knowledge, this is the first study to evaluate the impact of air pollution on behavioral development in schoolchildren using both indoor and outdoor air pollution levels measured at schools. We found a consistent pattern of positive associations between exposure to indoor and outdoor EC, BC, and, to a lesser extent, NO_2_ and SDQ scores for general behavioral development. Noise was not associated with general behavioral development (i.e., SDQ) scores, but was positively associated with ADHD symptom scores. In contrast, TRAPs exposures were not associated with ADHD symptom scores.

TRAPs concentrations at school were positively associated with SDQ scores for general behavioral development in schoolchildren after taking into account levels of residential air pollution. These results persisted after we adjusted our final models by noise. The results remained unchanged after restricting the analysis to those children attending the same school since early in life. Our analytical strategy (also including subscales from the SDQ and ADHD-DSM-IV in the further analysis) allowed us to conduct several comparisons. We are not aware of any available report on the negative impacts of exposure to air pollution (EC, BC, and NO_2_) during childhood on general behavioral development assessed by SDQ scores; therefore, it is not possible to compare our findings with those of others. However, our findings are consistent with several previous observations assessing air pollution early in life. Hyperactivity symptom scores at age 7 years were higher in association with elemental carbon exposures during the first year of life in a cohort of U.S. children (Newman et al. 2013). In a cross-sectional study conducted in India (Siddique et al. 2011), children living in urban areas (with higher concentrations of PM_10_) showed a 4-fold rise in the prevalence of ADHD (assessed using the ADHD-DSM-IV list) compared with children living in rural areas. ADHD symptom scores were not positively associated with TRAPs exposures in our cross-sectional study population. Unexpectedly, indoor EC exposure was negatively associated with ADHD symptom scores when adjusted for noise. We do not have a causal explanation for this finding, which may have been due to chance or bias.

A number of underlying mechanisms have been hypothesized for the observed negative associations between TRAPs and brain development (Block et al. 2012). Neuroinflammation implicating microglia has been proposed to play a central role (Block and Calderón-Garcidueñas 2009; Hanisch and Kettenmann 2007). In two studies conducted in Mexico, autopsies of 35 healthy children and young adults living in Mexico City (considered as exposed group) and about 12 healthy children and young adults living in a low-polluted town (considered as control group) were evaluated. Compared with the control group, the exposed group showed a significant up-regulation of some important inflammatory genes in several brain regions was found, as well as disruption of the blood–brain barrier, endothelial activation, oxidative stress, inflammatory cell trafficking, and accumulation of ultrafine particles in the respiratory nasal epithelium, olfactory bulb neurons, and the endothelium and basement membranes of olfactory bulb arterioles (Calderón-Garcidueñas et al. 2008, 2010). It has also been suggested that exposure to diesel exhaust particles may induce apoptosis of dopaminergic neurons in the presence of microglia (Block et al. 2004).

In our study, 74.4% of children were exposed to levels of noise greater than the 35dB recommended for teaching environments (Berglund et al. 1999). We detected a positive association between noise exposure at school and ADHD symptomatology. In the multi-exposure models, we detected some evidence of confounding of noise associations by TRAPs. The available evidence on the impact of noise on behavioral development is limited. In two studies, the authors found increased SDQ hyperactivity subscale scores associated with aircraft noise at school in children 9–10 years old (Crombie et al. 2011; Stansfeld et al. 2009). In the most recent study, the authors reported an increased SDQ hyperactivity score associated with road traffic noise exposure at home in children 10 years old (Tiesler et al. 2013). In our study population noise exposure was positively associated with ADHD symptom scores (ADHD-DSM-IV), but not with the SDQ hyperactivity score.

A number of mechanisms might contribute to associations between noise exposure and ADHD symptomatology (Stansfeld et al. 2005). These include noise annoyance (when noise interferes with the daily activities—that is, teaching sessions—and usually accompanied by negative responses), increased arousal, and frustration (Basner et al. 2014; Evans 2006). It has also been suggested that exposure to high levels of noise may produce externalizing symptomatology (i.e., ADHD symptoms) rather than internalizing symptomatology (i.e., depressive symptoms) as a consequence of increased arousal (Haines et al. 2001).

The results of the present study should be interpreted in the context of its limitations. We could not confirm that exposure preceded the outcomes given the cross-sectional design of our study. There was a short interval between measurement of TRAPs and the assessment of behavioral development. BREATHE measurements (made at the school level) were deseasonalized according to annual levels during the same year (2012) that the questionnaires were filled out. The LUR models were developed on ESCAPE measurements conducted in 2009, before the start of the BREATHE project. The generalizability of our findings may have been affected by selection bias in that children who participated in BREATHE were different from those who did not participate with respect to maternal educational level. About 60% of mothers in our study population had a university degree—higher than the regional average of 50% among women between 25 and 39 years old living in Barcelona (Ajuntament de Barcelona 2014). Although ADHD has been shown to be more prevalent among SES-disadvantaged groups (Russell et al. 2014), the prevalence of ADHD cases in our sample (6.5%) was similar to the prevalence of ADHD in Spain (6.8%) (Catalá-López et al. 2012). Conversely, the sex distribution among the BREATHE participants (50%) was very similar to the regional average in the same age group (5–9 years) and therefore increases the generalizability of our results. Moreover, the Urban Vulnerability Index of the schools was not associated with the school participation rate (Spearman’s correlation coefficient = –0.09, *p* = 0.61), which might suggest that SES was less likely to be a major predictor of participation. Another limitation might be the potential for residual SES confounding (Klassen et al. 2004; Russell et al. 2014). However, we adjusted our analyses for indicators of SES at both individual (maternal education) and area (SES vulnerability index) levels, and adjusting for additional SES variables did not result in any notable change in associations (data not shown). As in previous studies, the quality of the assessment of noise exposure may have been limited. Because special attention is given to noise levels during teaching sessions, when noise may be most detrimental (Basner et al. 2014), we examined road traffic noise exposure in classrooms. This represented an improvement over previous studies, which have generally relied on a single modeled or measured outdoor noise level in front of the school. Our noise measurements were each made in a single classroom in each school. However, in getting closer to measuring the true personal indoor environment, our assessment is also subject to exposure misclassification because of the high dependency of classroom street orientation on the actual noise exposure.

In our study population, SDQ scores were positively associated with TRAPs exposures, whereas ADHD-DSM-IV scores were positively associated with noise exposure. In our study, SDQ was rated by parents, whereas ADHD-DSM-IV questionnaire was rated by teachers. This approach does not allow us to compare the results obtained from the two scales. The use of two scales may, however, provide better comprehensive information on behavioral development. Whereas ADHD-DSM-IV is a questionnaire used for in-depth assessment of ADHD symptomatology, the SDQ is a screening questionnaire used for detecting general behavioral developmental problems including hyperactivity and attentional problems as only one of several outcomes. In addition, it has been suggested that the combination of parents’ and teachers’ reports is more sensitive in detecting behavioral problems than either alone when compared with independent psychiatric assessments (Goodman et al. 2000). Results from previous studies suggested that parents reported behavioral problems more frequently and of greater severity, and therefore the agreement between parents and teachers has been considered as poor to moderate (Dirks et al. 2011; Gomez 2007).

Estimated associations between TRAPs and the SDQ total difficulties score were statistically significant and consistent, but the magnitude of the associations was small. Adjusted mean ratios used to estimate associations between behavior scores and the exposures suggest a 7–8% increase in scores with IQR increases in TRAPs, where each score can be considered a “count” of prevalent symptoms, and the median total difficulties score was 8 (range, 0–32). Therefore, the results presented in this study could have a non-negligible societal impact given the link between the total difficulties score of SDQ and worse child mental health (Vostanis 2006). A similar explanation might be applied to the observed association between noise in schools and ADHD symptomatology.

## Conclusion

The results of this study suggest that higher levels of TRAPs (EC, BC, and NO_2)_ at school are associated with worse general behavioral development in schoolchildren. We also observed increased ADHD symptomatology associated with noise levels at school. Further assessments of the schools’ air quality, noise level, and child behavioral development are warranted to assess the temporality of the potential causal relationship. If confirmed, the public health implications of these findings would be of great importance considering the substantial personal and societal burden accompanying such behavioral problems.

## Supplemental Material

(404 KB) PDF
