# Supplementary material for: Traffic-Related Air Pollution, Noise at School, and Behavioral Problems in Barcelona Schoolchildren: A Cross-Sectional Study
Source: Environ Health Perspect. 2015 Aug 4;124(4):529–35. doi: 10.1289/ehp.1409449 (PMC4829987; doi:10.1289/ehp.1409449)
Supplement: (404 KB) PDF [file ehp.1409449.s001.acco.pdf]

Environ Health Perspect
DOI: 10.1289/ehp.1409449

Note to Readers: *EHP* strives to ensure that all journal content is accessible to all readers. However, some figures and Supplemental Material published in *EHP* articles may not conform to 508 standards due to the complexity of the information being presented. If you need assistance accessing journal content, please contact ehp508@niehs.nih.gov. Our staff will work with you to assess and meet your accessibility needs within 3 working days.

Supplemental Material

Traffic-Related Air Pollution, Noise at School, and Behavioral Problems in Barcelona Schoolchildren: A Cross-Sectional Study

Joan Forns, Payam Dadvand, Maria Foraster, Mar Alvarez-Pedrerol, Ioar Rivas, Mònica López-Vicente, Elisabet Suades-Gonzalez, Raquel Garcia-Esteban, Mikel Esnaola, Marta Cirach, James Grellier, Xavier Basagaña, Xavier Querol, Mònica Guxens, Mark J. Nieuwenhuijsen, and Jordi Sunyer

Table of Contents

Table S1. Bivariate analysis between SDQ total difficulties score and ADHD-DSM-IV and sociodemographic characteristics at school level (n=39)

Table S2. Multilevel Variance Inflation Factors (MVIFS), parameter estimates, standard errors, and t-values for the multilevel model

Table S3. Effect modification of noise in the association between SDQ total difficulties score and ADHD symptomatology (from ADHD-DSM-IV) and TRAPs exposure at school (µg/m^3) as continuous variable (based on an IQR increase)

Table S4. Adjusted mean ratio (aMRs and 95% CIs) of SDQ total difficulties score and ADHD symptomatology (from ADHD-DSM-IV) for black carbon exposure at school (µg/m^3) and at home address (µg/m^3) as continuous variable (based on an IQR increase)

Table S5. Adjusted mean ratio (aMRs 95% CIs) of SDQ total difficulties score and ADHD symptomatology (from ADHD-DSM-IV) for TRAPs exposure at school (µg/m^3) and noise at school (dB) as continuous variable (based on an IQR increase) restricted to these children attending the same school since 3 years and before (n=2.192)

Figure S1. Adjusted mean ratio (aMRs 95% CIs) of SDQ subscales (emotion symptoms, conduct disorders, hyperactivity/inattention, peer relationship problems and prosocial

behavior) for indoor and outdoor TRAPs exposure at school (μg/m3) and noise at school (dB) as continuous variable (based on an IQR increase):

Figure S2. Adjusted mean ratio (aMRs 95% CIs) of ADHD-DSM-IV list (Inattention and hyperactivity) for indoor and outdoor TRAPs exposure at school (μg/m3) and noise at school (dB) as continuous variable (based on an IQR increase):

Table S1. Bivariate analysis between SDQ total difficulties score and ADHD-DSM-IV and sociodemographic characteristics at school level (n=39):

Variables	Total problems (SDQ)				ADHD symptomatology (DSM-IV)			
	25th	50th	75th	p-value	25th	50th	75th	p-value
Type of school								
Public (n=19)	8.06	9.03	10.00	0.017	6.71	8.48	9.77	0.465
Private (n=20)	7.28	7.80	8.75		6.85	7.69	9.10	
Vulnerability index at school level								
Low (n=15)	7.01	7.77	8.31	0.084	6.78	8.31	9.02	0.477
Medium (n=12)	7.68	9.17	11.02		6.77	7.57	9.13	
High (n=12)	7.93	8.58	9.41		6.72	8.71	10.17	

p-value based on Kruskal-Wallis test

Table S2. Multilevel Variance Inflation Factors (MVIFS), parameter estimates, standard errors, and t-values for the multilevel model

Variable	MVIF	Coef.	Standard Error	T-value
EC indoor	1.35	-0.13	0.04	-3.33
Noise	1.30	0.20	0.05	4.44
EC outdoor	1.34	-0.07	0.04	-2.13
Noise	1.29	0.18	0.04	3.94
NO_2 indoor	1.45	0.04	0.04	0.90
Noise	1.40	0.11	0.05	2.49
NO_2 outdoor	1.37	-0.02	0.05	-0.42
Noise	1.40	0.14	0.04	3.26

Adjusted for sex, child's age, maternal education, urban vulnerability index at home address, air pollution (black carbon) at home, home tobacco use, urban vulnerability index at school and type of school.

Table S3. Effect modification of noise in the association between SDQ total difficulties score and ADHD symptomatology (from ADHD-DSM-IV) and TRAPs exposure at school (μg/m^3) as continuous variable (based on an IQR increase):

	Total difficulties score (SDQ)†					ADHD symptomatology (DSM-IV)‡				
	Low noise (<35dB)		High noise (≥35dB)			Low noise (<35dB)		High noise (≥35dB)		
	aMRs	CI95%	aMRs	CI95%	p for interaction	aMRs	CI95%	aMRs	CI95%	p for interaction
Indoor										
EC	1.18	(1.04, 1.33)**	1.05	(0.99, 1.12)	0.100	0.95	(0.73, 1.22)	0.94	(0.86, 1.02)	0.448
NO_2	1.04	(0.91, 1.19)	1.01	(0.94, 1.09)	0.563	0.93	(0.72, 1.19)	1.11	(1.02, 1.22)*	0.034
Outdoor										
EC	1.11	(1.00, 1.24)	1.07	(1.02, 1.13)*	0.269	0.96	(0.78, 1.18)	0.98	(0.91, 1.05)	0.276
NO_2	1.08	(0.96, 1.23)	1.06	(0.99, 1.15)	0.434	1.03	(0.82, 1.29)	1.04	(0.94, 1.16)	0.311

aMRs = adjusted Mean Ratios.

· Single-exposure models including TRAPs (EC and NO_2) were adjusted for sex, child's age, maternal education, urban vulnerability index at home address, air pollution (black carbon) at home, home tobacco use, urban vulnerability index at school and type of school.

†Including school as random effect

‡Including teacher as random effect

*p-value<0.05; **p-value<0.001

Table S4. Adjusted mean ratio (aMRs and 95% CIs) of SDQ total difficulties score and ADHD symptomatology (from ADHD-DSM-IV) for black carbon exposure at school (μg/m^3) and at home address (μg/m^3) as continuous variable (based on an IQR increase):

	Total difficulties score (SDQ)[a]			ADHD symptomatology (DSM-IV)[b]		
	aMRs	CI95%		aMRs	CI95%	
Outdoor						
BC at school	1.06	(1.02,	1.10)*	0.98	(0.93,	1.04)
BC at home address	1.01	(0.99,	1.04)	0.98	(0.95,	1.03)

aMRs = adjusted Mean Ratios.

Models including TRAPs (BC) were adjusted for sex, child's age, maternal education, urban vulnerability index at home address, home tobacco use, urban vulnerability index at school and type of school.

[a] Including school as random effect
[b] Including teacher as random effect

*p-value<0.05; **p-value<0.001

Table S5. Adjusted mean ratio (aMRs 95% CIs) of SDQ total difficulties score and ADHD symptomatology (from ADHD-DSM-IV) for TRAPs exposure at school (µg/m^3) and noise at school (dB) as continuous variable (based on an IQR increase) restricted to these children attending the same school since 3 years and before (n=2.192):

**Variables**	**EC and Noise**		**NO_2 and Noise**	
	**EC indoor**<br>**IQR = 1.01** µg/m^3<br>**EC outdoor**<br>**IQR = 0.86** µg/m^3	**Noise**<br>**IQR = 7.60 dB**	**NO_2 indoor**<br>**IQR = 21.01** µg/m^3<br>**NO_2 indoor**<br>**IQR = 22.26** µg/m^3	**Noise**<br>**IQR = 7.60 dB**
**Total difficulties score (SDQ)[a]**				
Indoor				
Single-exposure	1.07 (1.02, 1.13)**	1.01 (0.95, 1.07)	1.02 (0.96, 1.08)	1.01 (0.95, 1.07)
Multi-exposure	1.08 (1.03, 1.15)**	0.97 (0.91, 1.03)	1.02 (0.95, 1.09)	1.00 (0.94, 1.07)
Outdoor				
Single-exposure	1.07 (1.02, 1.11)**	1.01 (0.95, 1.07)	1.07 (1.01, 1.14)*	1.01 (0.95, 1.07)
Multi-exposure	1.07 (1.03, 1.13)**	0.97 (0.92, 1.04)	1.08 (1.01, 1.17)*	0.98 (0.91, 1.04)
**ADHD symptomatology (DSM-IV)[b]**				
Indoor				
Single-exposure	0.94 (0.87, 1.03)	1.23 (1.11, 1.36)**	1.05 (0.96, 1.16)	1.23 (1.11, 1.36)**
Multi-exposure	0.87 (0.80, 0.95)**	1.33 (1.19, 1.47)**	0.95 (0.85, 1.06)	1.27 (1.14, 1.43)**
Outdoor				
Single-exposure	0.98 (0.92, 1.06)	1.23 (1.11, 1.36)**	1.03 (0.94, 1.14)	1.23 (1.11, 1.36)**
Multi-exposure	0.93 (0.86, 1.01)	1.29 (1.16, 1.44)**	0.94 (0.85, 1.05)	1.27 (1.14, 1.42)**

- Single-exposure models including TRAPs (EC and NO2) were adjusted for sex, child's age, maternal education, urban vulnerability index at home address, air pollution (black carbon) at home, home tobacco use, urban vulnerability index at school and type of school.

- Single-exposure models including noise were adjusted for sex, child's age, maternal education, urban vulnerability index at home address, traffic noise annoyance at home, home tobacco use, urban vulnerability index at school and type of school.

• Multi-exposure models including TRAPs and noise were adjusted for sex, child's age, maternal education, urban vulnerability index at home address, air pollution (black carbon) at home, traffic noise annoyance at home, home tobacco use, urban vulnerability index at school and type of school.

[a] Including school as random effect
[b] Including teacher as random effect

*p-value<0.05; **p-value<0.001

Figure S1. Adjusted mean ratio (aMRs 95% CIs) of SDQ subscales (emotion symptoms, conduct disorders, hyperactivity/inattention, peer relationship problems and prosocial behavior) for indoor and outdoor TRAPs exposure at school (µg/m3) and noise at school (dB) as continuous variable (based on an IQR increase):

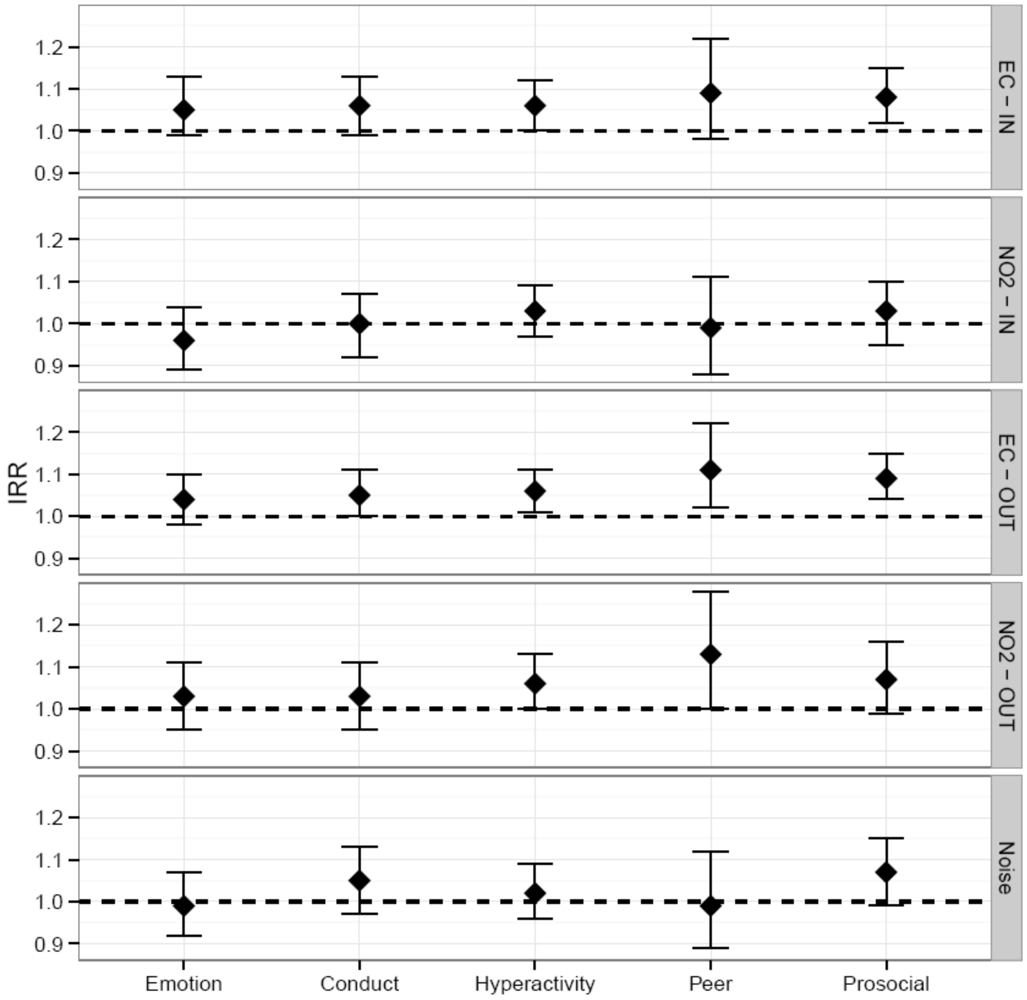

Footnotes:

· Models including TRAPs (EC and NO_2) were adjusted for sex, child's age, maternal education, urban vulnerability index at home address, air pollution (black carbon) at home, home tobacco use, urban vulnerability index at school and type of school.

· Models including noise were adjusted for sex, child's age, maternal education, urban vulnerability index at home address, traffic noise annoyance at home, home tobacco use, urban vulnerability index at school and type of school.

· Models using SDQ subscales also included school as random effect.
· Models using ADHD-DSM-IV subscales also included teacher as random effect.

Emotion=Emotional symptoms from SDQ; Conduct=Conduct disorders from SDQ; Hyperactivity=Hyperactivity/Inattention from SDQ; Peer=Peer relationship problems from SDQ;
Prosocial=Prosocial behavior from SDQ.

Figure S2. Adjusted mean ratio (aMRs 95% CIs) of ADHD-DSM-IV list (Inattention and hyperactivity) for indoor and outdoor TRAPs exposure at school (µg/m3) and noise at school (dB) as continuous variable (based on an IQR increase):

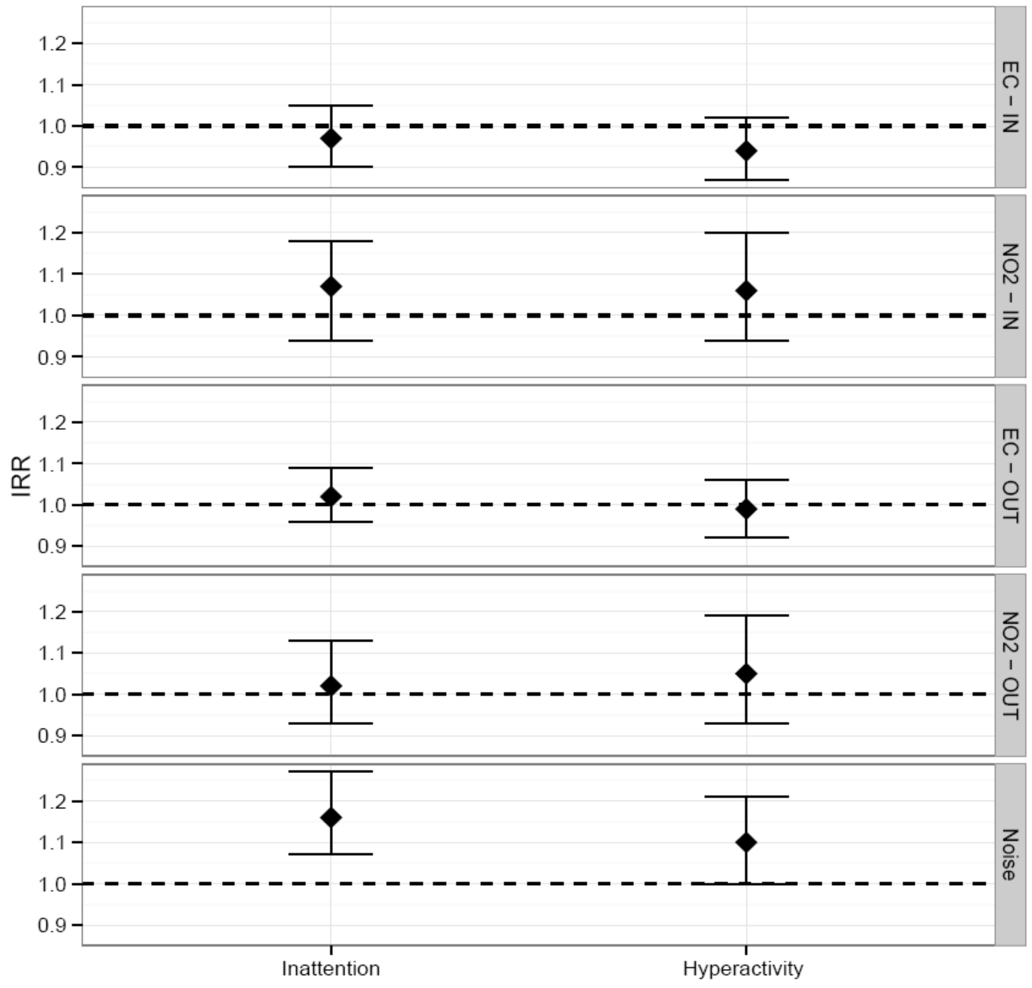

Footnotes:

· Models including TRAPs (EC and NO_2) were adjusted for sex, child's age, maternal education, urban vulnerability index at home address, air pollution (black carbon) at home, home tobacco use, urban vulnerability index at school and type of school.

· Models including noise were adjusted for sex, child's age, maternal education, urban vulnerability index at home address, traffic noise annoyance at home, home tobacco use, urban vulnerability index at school and type of school.

· Models using SDQ subscales also included school as random effect.
· Models using ADHD-DSM-IV subscales also included teacher as random effect.

Inattention=Inattention scale from ADHD-DSM-IV list.
Hyperactivity=Hyperactivity scale from ADHD-DSM-IV list.
